# Induced Effects of Sodium Ions on Dopaminergic G-Protein Coupled Receptors

**DOI:** 10.1371/journal.pcbi.1000884

**Published:** 2010-08-12

**Authors:** Jana Selent, Ferran Sanz, Manuel Pastor, Gianni De Fabritiis

**Affiliations:** 1 Computer-Assisted Drug Design Laboratory, Research Unit on Biomedical Informatics, IMIM, Universitat Pompeu Fabra, Barcelona, Spain; 2 Integrative Biomedical Informatics Laboratory, Research Unit on Biomedical Informatics, IMIM, Universitat Pompeu Fabra, Barcelona, Spain; 3 Computational Biochemistry and Biophysics Laboratory, Research Unit on Biomedical Informatics, IMIM, Universitat Pompeu Fabra, Barcelona, Spain; University of Houston, United States of America

## Abstract

G-protein coupled receptors, the largest family of proteins in the human genome, are involved in many complex signal transduction pathways, typically activated by orthosteric ligand binding and subject to allosteric modulation. Dopaminergic receptors, belonging to the class A family of G-protein coupled receptors, are known to be modulated by sodium ions from an allosteric binding site, although the details of sodium effects on the receptor have not yet been described. In an effort to understand these effects, we performed microsecond scale all-atom molecular dynamics simulations on the dopaminergic D_2_ receptor, finding that sodium ions enter the receptor from the extracellular side and bind at a deep allosteric site (Asp2.50). Remarkably, the presence of a sodium ion at this allosteric site induces a conformational change of the rotamer toggle switch Trp6.48 which locks in a conformation identical to the one found in the partially inactive state of the crystallized human β_2_ adrenergic receptor. This study provides detailed quantitative information about binding of sodium ions in the D_2_ receptor and reports a possibly important sodium-induced conformational change for modulation of D_2_ receptor function.

## Author Summary

G-protein coupled receptors represent more than 50% of the current drug targets, hence playing a crucial role in drug discovery today. A deeper understanding of G-protein coupled receptor functioning and modulation will help in the development of new drugs that are able to interact with such systems in a more subtle way than simple agonists or antagonists. In the present work, we studied the energetics of sodium ions, which have been described to act as an allosteric regulator within the D_2_ receptor using long-time molecular dynamics simulations, in order to gain insight into the molecular mechanism by which they exert this effect. In our simulations, we observed how sodium ions are able to induce a conformational change of the Trp6.48, a molecular rotamer switch which is implicated in the activation mechanism of G-protein coupled receptors. This observation, never reported before, has interesting implications for the design of drugs able to interact in a proper way with D_2_ receptor in particular and GPCR in general.

## Introduction

G-protein coupled receptors (GPCR) are highly sophisticated signal transduction machines able to respond to extracellular stimulus by activating diverse intracellular signaling pathways. Recent research in this field is unveiling the complexity of the mechanisms involved, which are far from being understood in detail. Among these mechanisms, allosteric modulation plays a central role in the fine-tuning of signaling. Unfortunately, the experimental study of allosteric regulatory processes in GPCRs is difficult because contemporary techniques (X-ray crystallography [1]–[5], and FRET [6], [7]) are unable to provide structural information of sufficient spatial resolution and time scales for describing specific atomic-level aspects of allosteric interactions. In this scenario, computational methods like molecular dynamics (MD) simulation can be used to provide unique insight into some elusive aspects of the problem, by meeting the aforementioned requirements in terms of both spatial resolution and time scale needed. The present work describes the application of long (microsecond) MD simulations for exploring the effect of sodium ions in class A GPCRs and the mechanism involved in their role as allosteric modulators.

**Figure 1:**
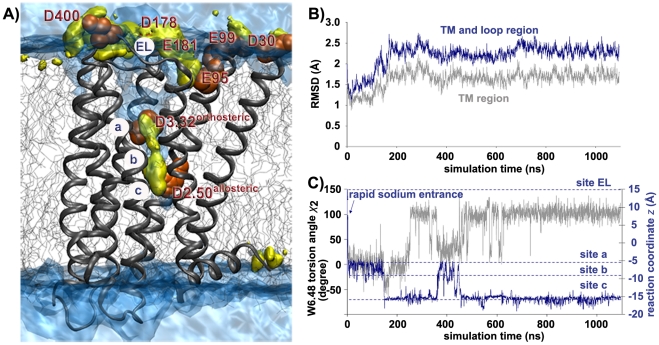
Sodium ion's pathway into the D_2_ receptor, receptor stability, and conformational change of the toggle switch Trp 6.48. (A) Volumetric map of sodium ions (yellow isosurface) within the D_2_ receptor at chemical potential μ = −4k_B_T relative to the bulk concentration of 150 mM NaCl showing the sodium ions binding sites. The ions move from the extracellular loop (EL) along negatively charged residues (orange spheres) towards the receptor interior (sites *a* to *c*) as computed from the ion concentration over 4.7 µs (MD2) of data. The volumetric map of water (blue isosurface) computed at μ = −0.5k_B_T relative to bulk water illustrates that part of the receptor interior is filled with water molecules including sites *a*, *b* and *c*. (B) The RMSD of the D_2_ receptor model embedded in a hydrated lipid bilayer over MD1; grey line: TM region, blue line: whole receptor including TM and loop region. (C) Depiction of the sodium ion's reaction coordinate *z* (blue line) and the Trp6.48 torsion angle χ_2_, (grey line) over MD1. Sodium transition from Asp3.32 (site *a*) to Asp2.50 (site *c*) induces a conformational change of the Trp6.48 rotamer switch.

Dopaminergic receptors are GPCRs, belonging to the class A family, which have been used as drug targets for the treatment of diverse central nervous system disorders (e.g. schizophrenia, Parkinson's disease). Allosteric modulation of dopaminergic GPCR by sodium ions has been extensively studied experimentally [8]–[13] and confirmed for the receptor subtypes in D_2_ and D_4_. It has been proposed that sodium ions bind in an allosteric site (Asp2.50, using residue numbering scheme of Ref. [14]) of the transmembrane (TM) region located below the orthosteric binding site (Figure 1A). The high conservation of Asp2.50 among GPCRs suggests its structural importance for the GPCR function. Presumably, the negatively charged carboxylic group of Asp2.50 interacts via electrostatic interaction with the positively charged sodium ion. Indeed, the introduction of uncharged residues in position 2.50 (Asp2.50Ala, Asp2.50Asn) produces sodium insensitivity in D_2_ and D_4_ [10], [15], demonstrating that the presence of a negative charge in the allosteric site is essential for maintaining sodium sensitivity. Neve *et al.* [16] proposed that the sodium binding site is a pyramidal hydrogen-bonding network formed by Asp2.50, Ser3.39, Asn7.45, and Ser7.46, with a sodium ion occupying the centre. However, and in spite of the experimental information available, the molecular mechanism of the allosterically sodium-induced modulation on GPCR activation remains unknown.

In an effort to elucidate the mechanism of the sodium-induced effect, we have computationally investigated the mobility of sodium ions in the sodium-sensitive D_2_ receptor. All-atom molecular dynamics (MD) simulations of the D_2_ receptor were performed to analyze more than 6 µs of simulation data. This analysis comprises a single 1.1 µs long simulation (MD1), one hundred 50 ns simulations (MD2) and biased metadynamics simulations (MD3) to compute accurate two-dimensional free energy profiles [17], [18] (see Table 1).

**Table 1:** Details of the performed MD simulations.

Name	No. of Runs	Description
MD1	1	Single molecular dynamics trajectory of 1.097 µs using a GPU workstation
MD2	100	Multiple trajectories of approximately 50 ns/each on GPUGRID
MD3	25	Metadynamics runs of 14 ns/each on GPUGRID

Molecular dynamics simulations were performed using ACEMD [36], [37], a new generation of molecular dynamics software which performs all the calculations on graphical processing units (GPUs) instead of standard CPUs, as well as GPUGRID.net (http://www.gpugrid.net) a globally distributed volunteer computing project [38], [39] harnessing the power of graphics cards for computation all around the globe (see Supporting Information for further details).

The simulation of the sodium ion's trajectory into the D_2_ receptor reveals a sodium ion entrance from the extracellular side of the receptor, which is followed by its threading into a pyramidal hydrogen-bonding network [16] near the allosteric binding site (Asp2.50) deep inside the receptor. Most remarkably, the analysis of all these trajectories showed a correlation between the position of the sodium and the conformation of the Trp6.48 side chain. This residue is part of the so called rotamer switch, a set of residues which are known to undergo major conformational change upon GPCR activation [19], [20], [21]. Kobilka and co-workers propose based on experimental data that the conformational state of the rotamer switch Trp6.48 is affected by ligands in the orthosteric binding site and is linked to a particular receptor response (agonists, partial agonists, neutral antagonist, inverse agonist) [22], [23]. Our study shows that, for dopaminergic receptors, the conformational state of the rotamer switch Trp6.48 can also be modulated from an allosteric site by sodium ions locking Trp6.48 in a conformational state identical to the partially inactive structure of the experimentally derived β_2_ adrenergic receptor [1].

## Results/Discussion

Recent advances in GPCR engineering and X-ray crystallography techniques has allowed the resolution of the structures of GPCRs closely-related phylogenetically to the D_2_ receptor (PDB ID codes: 2RH1 and 3D4S, 2VT4, 3EML). Superimposition of these templates shows larger structural deviations in the intra- and extracellular loop regions whereas high structural similarity is found in the TM region [24], indicating that the topology of the TM region is highly conserved among aminergic GPCRs.

In our study, the high resolution X-ray structure of the β_2_ adrenergic receptor (PDB entry 2RH1, resolution 2.40 Å) was selected as a template for the D_2_ receptor modeling (Figure 1A). The TM region, which is the major focus of our study, has a sequence homology of about 42% (calculation based on the PAM 250 scoring matrix), which is above the suggested threshold of 30% acceptable for transmembrane models of membrane proteins [25]. The Asp2.50 residue has been modeled in a deprotonated form as indicated by the predicted protonation state of the carboxylic acid side chain of Asp2.50 for a pH value at 7.4 (for details see Supporting Information). The predicted protonation state is in agreement with experimental studies which show that Asp2.50 is a putative sodium binding site with a deprotonated Asp2.50 side chain. In order to allow a full structural relaxation of the obtained homology model, the D_2_ receptor model is embedded in a realistic membrane bilayer environment (hydrated POPC, palmitoyloleoyl-phosphatidylcholine) and unconstrained MD simulations with the CHARMM forcefield [26] are carried out under physiological ionic strength conditions. The root mean square deviation (RMSD) values of the D_2_ receptor model over the course of a 1.1 µs molecular dynamics simulation (MD1) indicate that the structural relaxation of the receptor is reached after 190 ns (Figure 1B). Overall, the generated system proved to be very stable during more than 1 µs, with no major structural changes in the TM region of the D_2_ receptor and larger structural rearrangements in the more flexible loop regions (Figure 1B, and see Figure 1 in Text S1). Assessment of the final D_2_ receptor (snapshot at 1094 ns) relative to the initial D_2_ receptor model and the experimental X-ray crystal structures using global pairwise 3D structural alignments of 191 C-alpha atoms of their corresponding TM regions demonstrates that the primary 7TM topology is well maintained during simulation (RMSD<2 Å in TM region, see Table 1 in Text S1). Consequently, the D_2_ receptor model reflects a realistic ensemble of the helical arrangement, supporting the significance and physically realistic correctness of the generated system. Moreover, this structural conservation of the TM region shows that the larger helical movement associated with GPCR signaling is not seen in 1.1 µs simulation time.

### Pathway of Sodium Ions into the D2 Receptor

It has been proposed that sodium ions reach the allosteric binding site from the intracellular surface of the cell membrane [27]. However, the electrochemical gradient, which is directed from the extracellular toward the intracellular side, suggests a sodium entrance from the extracellular surface instead. Interestingly, in our generated model we observed that the equilibrated starting configuration of the D_2_ receptor/membrane system (equilibration time 20 ns) already shows an accumulation of sodium ions in the extracellular loop (EL) domain, with a preference for the negatively charged aspartates and glutamates. Chloride is found abundantly at positively charged residues in the cytosolic domain. This supports the hypothesis that sodium entrance is favored from the extracellular side. Recent MD simulation studies on the β_2_ adrenergic receptor/membrane system show a similar ionic distribution [28], and they are particularly in agreement with sodium binding sites in the extracellular loop region [29].

The computational experiment MD1, consisting in a long-time MD simulation over a single 1.1 µs trajectory, confirms that a sodium ion spontaneously penetrates the dopaminergic D_2_ receptor from the extracellular side (see Video S1 [30] of Supporting Information). The sodium transition into the receptor is reflected in Figure 1C by plotting the ion *z* coordinate (blue line) over the simulation time. The plot of sodium transition (Figure 1C) illustrates how the sodium ion enters rapidly from the extracellular side into the receptor towards sites *a* and *b*. The ion stays in these sites for about 180 ns before moving to site *c* where it stays for 200 ns. The ion returns to site *a*/*b* at 380 ns before it transits back to site *c* where it remains for the rest of the MD simulation (>600 ns). Although the ion enters the receptor extremely quickly, only the use of long-time simulations provides the complete trajectory of the sodium ions, which reach the final site *c* after 500 ns. Interestingly, we also observed that once one sodium ion has reached site *c*, a second sodium ion is able to enter the D_2_ receptor where it occupies the orthosteric site *a* (see Video S1 of Supporting Information).

In order to study in more detail the D_2_ sodium binding sites, we assessed the accumulated data of 100 independent runs of 50 ns leading to 4.7 µs of data (MD2). The volumetric map shown in Figure 1A represents the sodium chemical potential at μ = −4k_B_T compared to the bulk sodium concentration, providing detailed quantitative information about the ion's pathway into the D_2_ receptor. According to this map, the sodium enters the receptor in two steps.

In a first step, ions accumulate in the extracellular loop region showing a high population of negatively charged residues such as Asp400, Asp178 and Glu181. Subsequently, ions are guided by Glu95 into the receptor towards Asp3.32 (orthosteric site) and Asp2.50 (allosteric site). This observation suggests that small drug-like molecules might follow a similar two-steps pathway, since most ligands contain one or more basic nitrogen atoms and are positively charged at physiological conditions. In fact, site-directed mutagenesis experiments demonstrate that negatively charged residues in the extracellular surface are electrostatically involved in ligand binding. For instance, ligand binding is inhibited when Glu95, located in the extracellular loop 2, is mutated to cystein (Glu95Cys) and subsequently coupled to a positively charged sulfhydryl specific reagent, or even potentiated when coupled to a negatively charged reagent [11].

**Figure 2:**
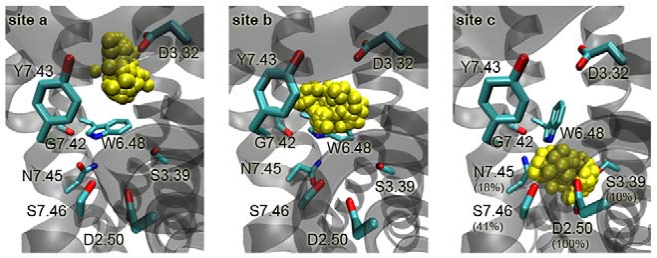
Sodium ion binding between the orthosteric and allosteric site. Distribution of sodium ions (yellow spheres) for binding sites *a* (*z*∼−5.0 Å, orthosteric), *b* (*z*∼−7.5 Å) and *c* (*z*∼−15.0 Å, allosteric) between Asp3.32 and Asp2.50. In site *c*, the percentage of sodium ion contacts in a 2.5 Å radius over time is given in brackets.

In the second step of the pathway, sodium ions visit not only the neighborhood of Asp3.32 (orthosteric binding site, or site *a*) and of Asp2.50 (allosteric binding site or site *c*) but also a location in the middle which is called site *b*. A similar analysis using bulk water (blue isosurface, Figure 1A) shows that the aforementioned binding sites *a*, *b* and *c* can be solvated by water molecules, a result which is consistent with the water molecules detected in the X-ray crystal structure of the β_2_ adrenergic receptor (see Figure 2 in Text S1).

### Sodium Binding Sites in the TM Region

In order to obtain a clearer picture of the sodium interaction binding sites *a*–*c*, described above, the accumulated data of the simulation MD2 were further structurally assessed (Figure 2). In site *a* (*z*∼−5.0 Å), the partial dehydration of the bound sodium ion and the receptor allows the formation of a salt bridge with the carboxylate group of Asp3.32, as shown in Figure 2. At location *b*, rehydration of the ion and the protein side chain takes place. At this point, direct contacts between receptor and the ion are very limited due to the recovered ion's hydration shell. The hydrated sodium ion occupies the space between Asp3.32, Gly7.42, Trp6.48, and Tyr7.43 as illustrated in Figure 2 for site *b* (*z*∼−7.5 Å). The last binding location *c* is favoured by the electrostatic attraction between the positively charged bound sodium ion and the negatively charged Asp2.50. Both, the sodium ion and the carboxylate group of Asp2.50 partially dehydrate before forming a salt bridge, as depicted in Figure 2 for site *c* (*z*∼−15.0 Å). In position *c*, the sodium ion is stabilized by a hydrogen-bonding network which involves interactions with Ser3.39, Asn7.45, Ser7.46 as well as the charge neutralizing interaction with Asp2.50.

Computation of the frequency for sodium ion contacts in a radius of 2.5 Å in site *c* reveals strong interactions with Asp2.50 (100% of times) and Ser7.46 (41%), and weaker interactions with Asn7.45 (18%) and Ser3.39 (10%) (Figure 2, site *c*). The observed binding location *c* around Asp2.50 is in agreement with site-directed mutagenesis studies which explore Asp2.50 as possible allosteric sodium site in sodium-sensitive receptor [10], [15].

### Mechanism of the Sodium-Induced Effect

**Figure 3:**
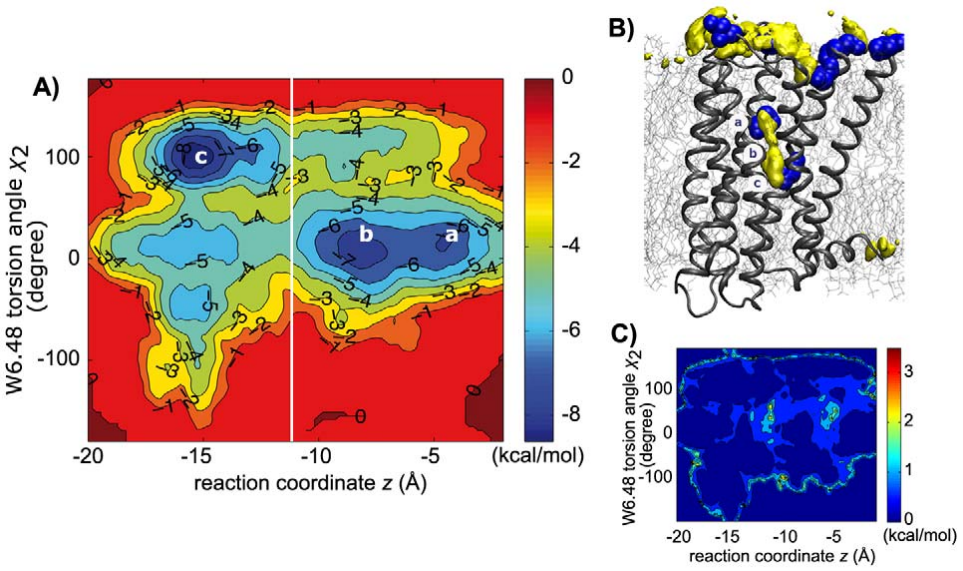
Free energy profile of sodium binding. (A) Two-dimensional free energy profile for (*z*,*χ_2_*) reaction coordinates, where *z* is the *z* coordinate of the bound sodium ion and *χ_2_* is the dihedral angle (CA-CB-CG-CD1) of tryptophan Trp6.48. The binding site *a*, *b*, *c* of the receptor show the pathway for sodium in the receptor and the locking of the tryptophan into the 100 degrees position (inactive state). The vertical white line shows the approximate position of Trp6.48. (B) Location of binding sites *a*, *b*, *c* in the D_2_ receptor. (C) Associated absolute free energy error map of the free energy profile of (A) computed by 25 metadynamics runs of 14 ns each (MD3). All basins have sub-kcal/mol error while only higher hills in the free energy surface show more significant variations.

In the course of sodium transition from site *a* to *c*, the position of the ion correlates with conformational changes of the Trp6.48 side chain which involves rotation around the torsion angle χ_2_ (CA-CB-CG-CD1) while the torsion angle χ_1_ (N-CA-CB-CG) is hardly affected (about −68 degrees). Figure 1C shows the result of monitoring the Trp6.48 torsion angle χ_2_ (CA-CB-CG-CD1) over the complete 1.1 µs single trajectory simulation, together with the bound ion's reaction coordinate *z* (grey: χ_2_ torsion angle, blue: *z* ions reaction coordinate). While the sodium is located in sites *a* and *b*, the Trp6.48 adopts a torsion angle of 0 degrees, but as soon as the ion transits from site *b* to site *c* (180 ns), the Trp6.48 torsion angle changes to a final value of 100 degrees. This event reoccurs at around 400 ns and suggests that the position of the bound sodium and its transition from site *a* to *c* correlates with the Trp6.48 torsion angle χ_2_. This finding is highly interesting since it can be linked to the biophysically observed conformational events that occur during activation/inactivation of GPCRs. In this context, the Trp6.48 has been implicated as a molecular rotamer switch that undergoes a major conformational change upon GPCR activation [19], [20], [21]. In the process of GPCR activation, the Trp6.48 side chain has to rotate from a vertical position (inactive state) in which the nitrogen is directed to TM2 (gauche conformation, supported by X-ray crystallography 1–5) into a horizontal position (active state) in which the nitrogen is directed to TM5 (trans conformation, supported by electron crystallography [21] and computational predictions [31]). Hence, during the activation process the receptor traverses several intermediate states which involve rotation of the Trp6.48 around the torsion angles χ_2_ (CA-CB-CG-CD1) as well as χ_1_ (N-CA-CB-CG) (see Figure 3 in Text S1). In this context, the conformational state of Trp6.48 with a torsion angle of χ_2_ = 0 present in the equilibrated starting configuration can be related to such an intermediate state whereas the Trp6.48 torsion angle of χ_2_ = 100 which is adopted in our simulation at 1.1 µs corresponds to the inactive state of the receptor (Figure 1C).

With respect to the energy changes associated with these events, their kinetics (in the order of hundred nanoseconds) prevent us obtaining accurate estimates from a single microsecond simulation. The sampling provided by the aggregate 4.7 µs simulation data (MD2) is able to capture several crossing events but insufficient to give an accurate statistical description. As an alternative, we ran a series of biased simulations over a two-dimensional reaction coordinate space (*z*, χ_2_) formed by the *z* coordinate of the ion and the χ_2_ (CA-CB-CG-CD1) angle. We performed 25 independent well-tempered metadynamics [17] runs (MD3) using the PLUMED ACEMD plugin [32], averaging the results in order to obtain also an estimation of the error associated with the energy values. The map is shown in Figure 3A, where the binding sites of the ion are labeled *a*, *b*, and *c*. The error associated with the computed energies is shown in Figure 3C. For most of the basins, it is possible to reproduce a well converged free energy map with sub-kcal/mol errors (see Supporting Information for details on average and error calculations).

**Figure 4:**
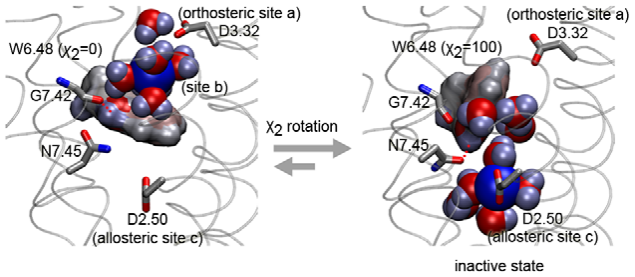
Mechanism of the sodium-induced effect on the rotamer switch (Trp 6.48). Sodium-induced effect is mediated by cation-Π interaction of the hydrated positively charged sodium ion and the negative charges of the Trp6.48 ring. The sodium ion, water molecules as well as the Trp6.48 are colored according to their partial charges (blue: positive, red: negative). Transition of a sodium ion from Asp3.32 (site *a*/*b*, χ_2_ = 0) to Asp2.50 (site *c*, χ_2_ = 100) induces a conformational change of Trp6.48 due to electrostatic interactions between the hydrated positively charged sodium ion and the negative charges of the indole ring of Trp6.48.

The results are also consistent with the previous unbiased simulations MD1 and MD2. The energetic map of Figure 3A shows a large basin formed by binding sites *a* (*z*∼−5.0 Å) and *b* (*z*∼−7.5 Å) for the ion before the ion passes the Trp6.48 residue located at about *z*∼−12.0 Å (white line). Within this basin the χ_2_ angle of the residue Trp6.48 is locked at 0 degrees with a barrier of 3 kcal/mol. Once the ion crosses the Trp6.48 residue passing an energetic barrier of approximately 3 kcal/mol, the binding site *c* is largely favored. At site *c*, the χ_2_ angle of Trp6.48 is now locked at 100 degrees with energetic barriers of 4 kcal/mol. The well depths of site *a*, *b* are similar, while site *c* seems to be favored by at least 1 kcal/mol. In order to move between site *b* and *c*, the bound ion has to overcome a barrier of about 3–4 kcal/mol, consistent with the slow kinetics shown by the microsecond scale simulation of Figure 1C. Consequently, the energetic map of sodium ion and the Trp6.48 conformation (Figure 3A) suggests that the presence of the sodium ion shifts the receptor equilibrium from receptor conformations with a Trp6.48 torsion angle of χ_2_ = 0 degree (sites *a*, and *b*) towards conformations with a torsion angle of χ_2_ = 100 degrees, corresponding to the inactive state when the ion is placed in site *c* (Figure 4).

The observed intermittent co-presence of a second sodium ion at the orthosteric binding site *a* (see Video S1 of Supporting Information), most likely contributes to the preferred site *c* of sodium binding. In the same manner, we can expect that the existence of an orthosteric dopaminergic agonist or antagonist in site *a* is compatible with sodium binding in site *c*.

Mechanistically, the Trp6.48 toggling is mediated by cation-Π interaction of the hydrated, positively charged sodium ion and the negative charges of the Trp6.48 ring. In site *b* (Figure 4, left), the water molecules of the hydrated sodium ion form hydrogen bonds with the Trp6.48 which then adopts a dihedral angle of χ_2_ = 0. This conformation is further stabilized by a hydrogen bond of Trp6.48 with the carboxyl group of the Gly7.42 backbone. As the hydrated sodium ion passes from site *b* to site *c* (Figure 4, right), Trp6.48 toggles (χ_2_ = 100). The inactive conformation is stabilized again by cation-Π interaction mediated by water molecules of the ion's hydration shell now in site *c*, and additionally by the formation of a stabilizing hydrogen bond to Asn7.45.

Locking the rotamer switch Trp6.48 in its inactive conformations could disfavor agonist binding to the Asp3.32 in the orthosteric site (Figure 1A). Indeed, it has been suggested that sodium ions are able to reduce agonist binding affinity by increasing the population of receptor conformations with low agonist affinity [9]. This finding is supported by experimental data which demonstrate that agonist binding is decreased in the presence of sodium ions [9], [33].

### Conclusions

The ability of sodium ions to regulate receptor functioning of dopaminergic GPCR from an allosteric site has been extensively studied in previous experimental work. Here, we show the results of MD simulations at the microsecond scale to investigate the sodium-induced effects on GPCRs, focusing on the dynamics and energetics of sodium ions in the D_2_ receptor. All-atom, unconstrained MD simulations show a two step binding of sodium ions to the dopaminergic D_2_ receptor. First, negatively charged residues (Asp400, Asp178, Glu181 and Glu95) in the extracellular surface are involved in forming a large favorable volume for sodium at the receptor entrance (Figure 1A) which is consistent with previous computational observations [28], [29]. Second, the sodium ion visits three binding locations (*a*–*c*) between Asp3.32 (site *a*) and Asp2.50 (site *c*). The computed energetics of the sodium ion binding indicate that site *c* is energetically favored over sites *a* and *b*.

The existence of the allosteric sodium binding site *c* is in agreement with experimental data [16]. Most importantly, the computational results indicate that sodium ions that transit from site *a* to *c* induce a conformational change acting like a fingertip toggling and locking the rotamer switch (Trp6.48) in its inactive state.

## Methods

For the realistic, three-dimensional structural molecular system, a D_2_ homology model was built based on the β_2_ adrenergic receptor (PDB ID 2RH1) according to a previously used protocol [34]. The highly flexible N-terminal (sequence 1–29) and intracellular loop 3 (sequence 221–365) were omitted since no adequate template exists and because the putative binding site of sodium is located at Asp2.50 in the seven transmembrane region. The D_2_ receptor model was inserted into a hydrated and pre-equilibrated POPC lipid bilayer membrane (membrane builder tool of the VMD version1.8.6) [35]. The initial system was equilibrated at 1 atm using the CHARMM force field [26] with rigid bonds, time step 2 fs, cutoff 9 Å, PME grid size of 80×80×96, Langevin damping 0.1/ps. After the initial equilibration phase, all the production runs were carried out using ACEMD [36], [37] at 300 K, in the NVT ensemble (damping factor 0.1/ps) to preserve the lipid cross section over long simulation times. Molecular images were produced using VMD [35]. Further details are provided in Supporting Information.

## Supporting Information

Text S1 Additional information.
(0.32 MB PDF)

Video S1 A 1.1 µs long-time MD simulation demonstrates that a sodium ion spontaneously penetrates the dopaminergic D_2_ receptor from the extracellular side. Once inside the receptor, the sodium ion provokes a conformational change of the rotamer switch Trp6.48. Moreover, in the presence of a sodium ion site c (below the rotamer switch Trp6.48), a second sodium ion is able to enter at times the D_2_ receptor where it occupies the orthosteric site a (above the rotamer switch Trp6.48).
(4.96 MB AVI)
