# Supplementary material for: Induced Effects of Sodium Ions on Dopaminergic G-Protein Coupled Receptors
Source: PLoS Comput Biol. 2010 Aug 12;6(8):e1000884. doi: 10.1371/journal.pcbi.1000884 (PMC2920834; doi:10.1371/journal.pcbi.1000884)
Supplement: Text S1 — Additional information. (0.32 MB PDF) [file pcbi.1000884.s001.pdf]

Induced Effects of Sodium Ions on Dopaminergic G-Protein Coupled Receptors

Jana Selent[1], Ferran Sanz[2], Manuel Pastor[2], and Gianni De Fabritiis[3*]*

[1]Computer-Assisted Drug Design Laboratory,[2]Integrative Biomedical Informatics Laboratory, [3]Computational Biochemistry and Biophysics Laboratory, GRIB, IMIM, Universitat Pompeu Fabra, C/ Dr. Aiguader 88, 08003, Barcelona, Spain

Supporting Information

Content

1. Protein preparation and homology modeling of the dopaminergic D_2 receptor

2. Generation of the dopaminergic D_2 receptor/POPC membrane system

3. Equilibration protocol of the D_2 receptor/POPC membrane system

4. Production runs

   - Single, long trajectory (MD1)

   - Volumetric Maps (MD2)

   - Metadynamics free energy calculations (MD3)

5. System stability

6. Receptor states during the activation process

7. References

1. Protein preparation and homology modeling of the dopaminergic D_2 receptor

D_2 receptor (accession P14416) was retrieved from the Swiss-Prot database [1]. The generalized numbering scheme proposed by Ballesteros and Weinstein [2] was used for residues belonging to helix regions of the G-protein coupled receptors (GPCR). The high flexible N-terminal (sequence 1-29) and intracellular loop 3 (sequence 221-365) were removed since no adequate template exists and due to the fact that the putative binding site of sodium is located at Asp2.50 in the seven transmembrane (TM) region. The shortened sequence was aligned with the ClustalX software [3, 4] using the PAM250 matrix and "gap open" and "gap elongation" penalties of 10 and 0.05, respectively. The resulting multiple sequence alignment was realigned with the crystal structure of the human $ß_2$ adrenergic G-protein coupled receptor (PDB entry 2RH1) [5, 6] introducing secondary structure information derived from the crystal structure to avoid gaps within the seven helical segments. The alignment was then manually refined to ensure a perfect alignment of the highly conserved residues of GPCR superfamily, according to Baldwin *et al.*[7]. Extension of each helix was contemplated by taking into account the experimental length of the 2RH1 helices and the sequence conservation. The conserved disulfide bond between residues C3.25 at the beginning of TM3 and the cysteine in the middle of the extracellular loop 2 (a feature highly common among GPCRs) was also created and kept as a constraint in the geometric optimization. 3D models were then built using the MODELLER suite of programs [8] which yielded 15 candidate models. From these candidates, the best structures according to the MODELLER objective function and to visual inspection were selected. Models with interruptions or gaps in the TM regions, as identified by visual inspection, were discarded. The resultant structures of the receptors were optimized using the Amber99 force field [9] applying the molecular modeling program MOE (Molecular Operating Environment; Chemical Computing Group). PROCHECK software [10] was used to assess the stereochemical quality of the minimized structures resulting in good quality parameters with an excellent distribution of Psi and Phi angles in the Ramachandran plot (over 90% of the residues are in the most favored regions).

2. Generation of the dopaminergic D_2 receptor/POPC membrane system

In order to place the D_2 receptor model into the bilayer membrane, a hole was generated in a pre-equilibrated palmitoyloleoylphosphatidylcholine (POPC) bilayer membrane (membrane builder tool of the VMD version1.8.6) by removing POPC molecules. Lipids which were in close contact with the protein atoms (<1 Å distance from any protein atoms) were deleted, and water molecules were added to the system. An all-atom model of the D_2 receptor was generated using the CHARMM27 force field and parameter set with the TIP3 water model. The protonation state of titratable groups was predicted for a pH value at 7.4 based on PROPKA [11] using the implemented prediction tool of the MOE package. Seven aspartates located in the extracellular surface and TM region were built in the unprotonated form. The putative sodium binding site Asp2.50 in the TM domain were modeled with unprotonated carboxylic acid side chains (negatively charged) as the experimentally suggested presence of a sodium ion in the receptor would compensate the negative charge of Asp2.50. Furthermore, six glutamate residues which are located in extra- and intracellular regions were modeled in an unprotonated form; five histidine residues were kept in their neutral form; all arginine and lysine residues were modeled in protonated form. The protein model had a total net charge of +8e, which is balanced with 27 sodium and 35 chloride ions in the aqueous layer of 11094 water molecules containing a phospholipids bilayer with 170 POPC (palmitoyloleoylphosphatidylcholine) molecules. The coordinates for water and ions were generated using the solvate and autoionize modules of VMD1.8.6.

3. Equilibration protocol of the D_2 receptor/POPC membrane system

The initial equilibration of the system was carried out with NAMD2.6, using rigid bonds, time step 2 fs, cutoff 9 Å, PME grid size of 80x80x96, Langevin damping 0.1/ps. The system was first minimized for 1000 steps, then thermalized for 80 ps at 300 K reinitializing the velocities every 20 ps. A positional restrain of 1 kcal/mol/$Å^2$ was applied to all heavy atoms of the receptor. The system was then equilibrated at 1 atm for 100 ps, while the restraints were progressively reduced to zero. A final run of 20 ns was performed to equilibrate the pressure during which the potential energy and volume of the simulation box

were monitored to assure equilibration. After 15 ns both quantities remain stable. The final configuration box (78.76,78.76,94.21) Å was further equilibrated at constant temperature (NVT) using ACEMD for further 20 ns at 300 K, with a time step equal 2 fs, cutoff 9 Å, switch distance 7.5 Å, rigid bonds and a PME grid size of 80x80x96.

4. Production runs

For the production runs we have used ACEMD running on local workstations equipped with 4 Nvidia GPUs each and the distributed computing project GPUGRID. ACEMD is a new generation molecular dynamics software which runs exclusively on graphics processing units (GPUs) at the equivalent speed of hundred of standard processors [12, 13] (see also reference [14] for a review of the impact of accelerator processors and graphics cards on computational biology). GPUGRID is a distributed computing project based on the use of graphics processors around the globe [15] and the ACEMD molecular dynamics software [12]. At the time of these runs GPUGRID comprised of over 4000 GPUs for a total peak performance of 1 petaflop, and a sustained performance of 150 Teraflops.

After the initial equilibration phase and for all the production runs the simulation are run in the NVT ensemble, with the same parameters used for the equilibration but a timestep of 4 fs thanks to the use of the hydrogen mass repartition scheme implemented in ACEMD [16]. This elegant scheme allows for longer timesteps up to 4 fs by using the property that individual atom masses do not appear explicitly in the equilibrium distribution, therefore changing them only affects the dynamical properties of the system (marginally) [16] but not the equilibrium distribution. The lipid cross section is at equilibrium 64.6 Å^2/lipid as estimated from the equilibrated configuration and in good agreement with the experimental cross section for POPC [17]. As we run the long simulations in the NVT ensemble this cross section is maintained even during long runs and large sampling.

We have reported in this work three final runs according to Table 1 of the main manuscript: MD1, MD2, MD3.

Single, long trajectory (MD1). A single run of 1.097 μs was performed on a workstation using ACEMD in parallel over three Nvidia 275 GPUs for a total time of 66 days of continuous computation. A movie of the entire simulation is available online at http://www.vimeo.com/7900216 and in Video S1.

Volumetric Maps (MD2). The second production run (MD2 in Table 1) consists of 100 independent runs of approximately 50 ns each for a total sampling of 4.7 μs which were successfully returned by the GPUGRID server. The trajectory dumping frequency was set to 50 ps to reduce data upload to the server. The entire calculations required a couple of months although occupying only a fraction of the capability of GPUGRID.

For the analysis we have first discarded the first 20 ns of each run to avoid any bias of the initial condition, sampling the trajectory every 100 ps. The concatenated trajectory data was then wrapped for the periodic boundary conditions centered around the protein and then aligned to the same initial configuration. The tool VolMap from VMD was used to produce the volumetric map for ions and water (atom radius of 1.0 Å) using a grid size resolution of 0.5 Å and computing the ion and water occupancy per grid cell. The volumetric map of sodium ions (yellow isosurface, Figure 1A) was computed for a chemical potential $\mu = -4k_BT$ which corresponds to an isosurface c = 0.002465 particle/ $Å^3$ based on: $\Delta\mu = -k_BT \log(c/c_{bulk})$, with the bulk ions concentration being 150 mM NaCl (c_{bulk} = 0.000090307 particle/ $Å^3$). The volumetric map of water (blue isosurface, Figure 1A) was computed for the chemical potential $\mu = 0.514k_BT$ which corresponds to an isosurface c = 0.20000 particle/ $Å^3$ relative to the concentration of water at 300 K, 1 ATM (c_{water} = 0.334438 particle/ $Å^3$).

Metadynamics free energy calculations (MD3). Metadynamics [18] is a biased dynamics technique widely used to improve sampling for free energy calculations over a set of multidimensional reaction coordinates which would not be sampled exhaustively with normal unbiased simulations. It is implemented in the molecular dynamics code ACEMD using the PLUMED plugin interface [19]. Metadynamics is used to complement the unbiased simulation. According to the unbiased simulations, a

two-dimensional reaction coordinate is chosen to represent the slow degrees of freedom: The first coordinate is the *z* position of the bound sodium ion, while the second one is the dihedral angle χ_2 of tryptophan W6.48 formed between atoms (CA-CB-CG-CD1). The metadynamics parameters are set to a Gaussian hill height of 0.2 kcal/mol with a spread of 0.2 Å for the *z* coordinate and 0.1 rad for the dihedral angle. The deposition rate is one hill every 2 ps and a well-tempered bias factor of 8. Small changes on these reference parameters did not seem to affect the results. In order to estimate not only the free energy, but also the error associated with it, we performed 25 independent metadynamics runs of 14 ns each on GPUGRID. Each run started from an equilibrated configuration with the sodium ion outside of the deep binding pocket of the receptor. The simulations were binned over 100 bins between -20 to -1 Å for the *z* coordinate and between $-\pi$ and π for the dihedral angle χ_2. The average free energy $W(z, \chi_2)$ is reconstructed as the average over the estimate of the number states $N(z, \chi_2) = \langle exp(-W_i(z, \chi_2)/k_BT\rangle_i$ of each individual metadynamics run, where $W_i(z, \chi_2)$ is the free energy determined by the individual metadynamics run i, and

$$W(z, \chi_2) = - k_BT \log (\langle exp(-W_i(z, \chi_2)/k_BT \rangle_i).$$

The error in the estimation of $W(z, \chi_2)$ is measured similarly by computing the standard deviation in the number of states $S(z, \chi_2) = \langle (exp(-Wi(z, \chi_2)/k_BT- N)^2\rangle^{1/2}$. The total free energy error is then estimated as $W^{err}(z, \chi_2) = - k_BT \log (N(z, \chi_2) - S(z, \chi_2)) + k_BT \log (N(z, \chi_2)+S(z, \chi_2))$, as the free energy difference between an error of a standard deviation in N.

5. System stability

The generated system is stable during 1.1 µs simulation (MD 1). First, no major structural changes in the TM region of the D_2 receptor occurred. As expected, larger structural rearrangements in the more flexible loop regions were observed (Figure 1) Second, comparison of the TM topology of the final D_2 receptor model relative to experimental X-ray crystal structures demonstrates that the primary 7TM topology is well maintained during simulation (RMSD < 2 Å in TM region, Table 1). Third, the binding pocket of the

D_2 receptor is filled with water molecules which mostly coincide with water molecules found in the X-ray crystal structure of the β_2 adrenergic receptor (see Figure 2).

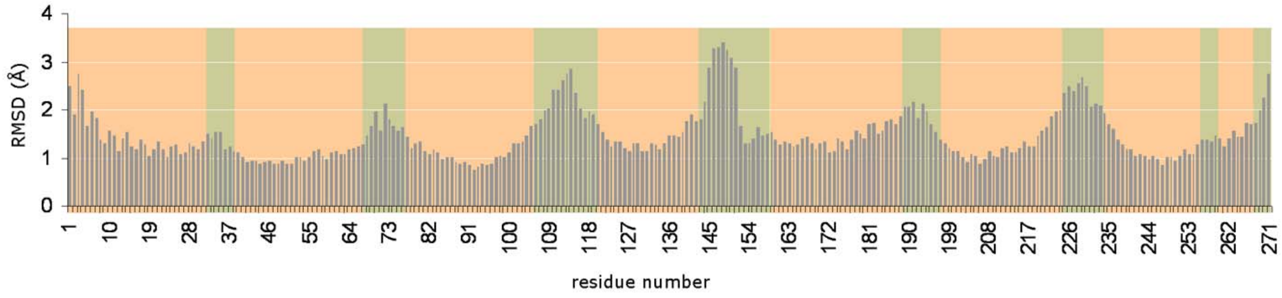

Figure 1. Fluctuation for each residue of the D_2 receptor model relative to the initial starting structure computed over 1.1 μs simulation time (MD1). Intra- and extracellular loop regions (green) are characterized by higher fluctuation than TM region (red) of the D_2 receptor model.

Table 1. Comparison of the TM topology of the final D_2 receptor model relative to experimental X-ray crystal structures using global pairwise 3D structural alignments of 191 C-alpha atoms of their corresponding TM regions

GPCR	PDB ID	RMSD
human β_2 adrenergic receptor in complex with (S)-carazolol	2RH1	0.000
human β_2 adrenergic receptor in complex with timolol	3D4S	0.359
turkey human β_1 adrenergic receptor in complex with cyanopindolol	2VT4	1.165
human A_{2A} adenosine receptor bound to ZM241385	3EML	1.907
**D_2 receptor model at 1.1 μs simulation time**	**-**	**1.864**

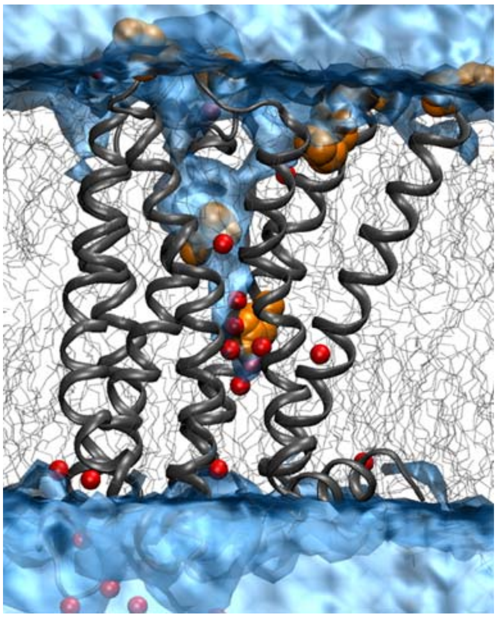

Figure 2. Volumetric map of bulk water (blue isosurface) computed over 4.7 µs simulation data (MD2) in superimposition with the X-ray crystal water of the β_2 adrenergic receptor (PDB ID 2RH1).

6. Receptor states during the activation process

In the process of GPCR activation, the rotamer switch Trp6.48 undergoes a major conformational change [20] and rotates from a vertical position in which the nitrogen is directed to TM2 (gauche (g) conformation) into a horizontal position in which the nitrogen is directed to TM5 (trans (t) conformation)[21].During this process the receptor has to undergo intermediate conformational states which involves rotation of the Trp6.48 around the torsion angles χ_2 (CA-CB-CG-CD1) as well as χ_1 (N-CA-CB-CG) (Figure 3).

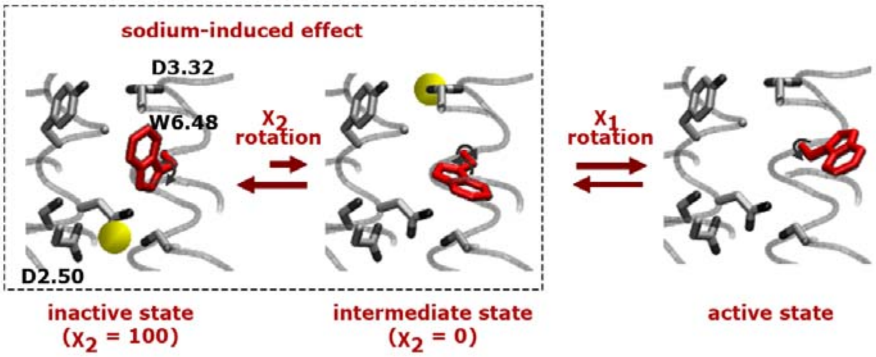

Figure 3. Receptor states during the activation process.

7. References

1. Boeckmann B, Bairoch A, Apweiler R, Blatter MC, Estreicher A, et al. (2003) The SWISS-PROT protein knowledgebase and its supplement TrEMBL in 2003. Nucleic Acids Res. 31: 365-370.

2. Ballesteros J, Weinstein H (1995) Integrated methods for the construction of three dimensional models and computational probing of structure function relations in G-protein coupled receptor. Methods Neurosci. 25: 366-428.

3. Thompson JD, Higgins DG, Gibson TJ (1994) CLUSTAL W: improving the sensitivity of progressive multiple sequence alignment through sequence weighting, position-specific gap penalties and weight matrix choice. Nucleic Acids Res. 22: 4673-4680.

4. Thompson JD, Gibson TJ, Plewniak F, Jeanmougin F, Higgins DG (1997) The CLUSTAL_X windows interface: flexible strategies for multiple sequence alignment aided by quality analysis tools. Nucleic Acids Res. 25: 4876-4882.

5. Cherezov V, Rosenbaum DM, Hanson MA, Rasmussen SG, Thian FS, et al. (2007) High-resolution crystal structure of an engineered human beta2-adrenergic G protein-coupled receptor. Science 318: 1258-1265.

6. Rasmussen SG, Choi HJ, Rosenbaum DM, Kobilka TS, Thian FS, et al. (2007) Crystal structure of the human beta2 adrenergic G-protein-coupled receptor. Nature 450: 383-387.

7. Baldwin JM, Schertler GF, Unger VM (1997) An alpha-carbon template for the transmembrane helices in the rhodopsin family of G-protein-coupled receptors. J.Mol.Biol. 272: 144-164.

8. Sali A, Blundell TL (1993) Comparative protein modelling by satisfaction of spatial restraints. J.Mol.Biol. 234: 779-815.

9. Cornell WD, Cieplak P, Gould IR, Merz KM, Ferguson DM, et al. (1995) A second generation force field for the simulation of proteins, nucleic acids, and organic molecules. J Am Chem Soc 117: 5179-5197.

10. Laskowski RA, MacArthur MW, Moss DS, Thornton JM (1993) PROCHECK: A program to check the stereochemical quality of protein structures. J. Appl. Crystallogr. 26: 283-291.

11. Li H, Robertson AD, Jensen JH (2005) Very fast empirical prediction and rationalization of protein pKa values. Proteins 61: 704-721.

12. Harvey MJ, Giupponi G, Fabritiis GD (2009) ACEMD: Accelerating Biomolecular Dynamics in the Microsecond Time Scale. Journal of Chemical Theory and Computation 5: 1632-1639.

13. Harvey MJ, De Fabritiis G (2009) An Implementation of the Smooth Particle Mesh Ewald Method on GPU Hardware. Journal of Chemical Theory and Computation 5: 2371-2377.

14. Giupponi G, Harvey MJ, De Fabritiis G (2008) The impact of accelerator processors for high-throughput molecular modeling and simulation. Drug Discov Today 13: 1052-1058.

15. GPUGRID.net. Available at: http://www.gpugrid.net.

16. Feenstra KA, Hess B, Berendsen HJC (1999) Improving efficiency of large time-scale molecular dynamics simulations of hydrogen-rich systems. Journal of Computational Chemistry 20: 786-798.

17. Lantzsch G, Binder H, Heerklotz H (1994) Surface area per molecule in lipid/C12En membranes as seen by fluorescence resonance energy transfer. Journal of Fluorescence 4: 339-343.

18. Laio A, Parrinello M (2002) Escaping free-energy minima. Proc. Natl. Acad. Sci. U.S.A 99: 12562-12566.

19. Bonomi M, Branduardi D, Bussi G, Camilloni C, Provasi D, et al. (2009) PLUMED: A portable plugin for free-energy calculations with molecular dynamics. Computer Physics Communications 180: 1961-1972.

20. Ruprecht JJ, Mielke T, Vogel R, Villa C, Schertler GFX (2004) Electron crystallography reveals the structure of metarhodopsin I. EMBO J 23: 3609-3620.

21. Bhattacharya S, Hall SE, Vaidehi N (2008) Agonist-induced conformational changes in bovine rhodopsin: insight into activation of G-protein-coupled receptors. J Mol Biol 382: 539-555.
